# Silver Nanowire-Amorphous Indium Zinc Oxide Composite Electrodes for Transparent Film Heaters

**DOI:** 10.3390/nano15241883

**Published:** 2025-12-15

**Authors:** Xingzhen Yan, Mengying Lyu, Ziyao Niu

**Affiliations:** 1 Key Laboratory of Architectural Cold Climate Energy Management, Ministry of Education, Jilin Jianzhu University, 5088 Xincheng Street, Changchun 130118, China; 2 Jilin Provincial Key Laboratory of Architectural Electricity & Comprehensive Energy Saving, Jilin Jianzhu University, Changchun 130118, China

**Keywords:** transparent conducting, silver nanowire, indium zinc oxide, heater

## Abstract

Transparent conductive films based on silver nanowire meshes have demonstrated significant potential as alternatives to conventional tin-doped indium oxide and fluorine-doped tin oxide thin films. However, these materials feature high junction resistance, poor damp heat (DH) stability, and weak mechanical adhesion to substrates, which are critical issues that must be addressed before any practical applications. In this paper, transparent conducting films composed of silver nanowire (AgNW) frameworks and amorphous indium zinc oxide (IZO) fillers were prepared by a spin-coating method. The AgNW-IZO composite films exhibited a higher conductivity and better DH stability and adhesion to substrates than that of their constituent parts alone. The lowest sheet resistance of the composite films was 3.3 ohm/sq with approximately 70% transparency in the visible spectrum. No degradation was observed after 8 months. The excellent DH stability and mechanical adhesion might facilitate applications of these AgNW-IZO composite films in optoelectronic devices. Furthermore, the composite electrode is shown to have potential as a transparent heater.

## 1. Introduction

Transparent conductive films (TCFs) are widely used as electrodes for various optoelectronic devices such as wearable electronics [1,2,3], light-emitting diodes (LEDs) [4,5,6,7], solar cells [8,9,10]. To date, indium tin oxide (ITO) and fluorine-doped tin oxide (FTO) are the most widely used TCF materials in industrial applications. Among the two, ITO exhibits the highest electrical conductivity and represents the most technologically mature option. However, the cost of pure ITO films has increased significantly over the past decade, driven by growing demand for flat-panel displays and solar cells, due to the limited natural abundance of indium in the Earth’s crust [11]. Furthermore, ITO is inherently brittle, which limits its compatibility with flexible substrates in optoelectronic applications [12]. To further enhance the photoelectric performance and mechanical stability, significant research attention has been directed toward alternative TCFs, such as silver nanowires (AgNWs) [13,14,15], carbon nanotubes [16,17], graphene [18], and conductive polymers [19]. Among these materials, AgNW meshes exhibit the highest electrical conductivity while maintaining high optical transparency (>85%) across a broad spectral range [20]. Furthermore, AgNWs-based transparent conductive films can be fabricated through cost-effective and scalable techniques, such as spin-coating and inkjet printing. However, the high resistance at nanowire-nanowire junctions, poor adhesion to substrates, and instability under harsh environmental conditions remain critical challenges that must be overcome to facilitate the broader application of such metal nanowires [21,22,23].

It is essential to increase the contact area at the junctions within the AgNW mesh to mitigate high contact resistance, thereby minimizing its adverse effects on carrier transport. Annealing and laser processing techniques can be utilized to promote fusion between nanowires to ensure robust and intimate interwire contact [12,24]. To address the challenges of weak adhesion between pure AgNW mesh electrodes and their substrates, as well as limited environmental stability, researchers have developed composite structural designs. Coating materials explored in these approaches include organic polymers, graphene-based materials, and metal oxides [14,25,26]. However, the conductivity of the composite electrode gradually deteriorates due to chemical erosion of the nanowires by residual species within the polymer matrix and inherent degradation mechanisms. Additionally, the inherently low electrical conductivity of graphene-based coating layers limits their ability to further reduce the overall resistance of the composite electrodes.

In this paper, we demonstrate a long-term stable TCF fabricated by combining a highly conductive AgNW mesh with conductive amorphous indium zinc oxide (IZO) by a simple spin-coating method. Both the AgNWs and IZO were prepared using solution-based processes, which could enable cost-effective and highly efficient large-scale production. The addition of IZO to form AgNW-IZO composite TCFs can enhance the film conductivity by providing more efficient electron conduction pathways. In addition, this composite film exhibits enhanced environmental stability and improved adhesion to the substrate, owing to the chemical inertness of oxide compounds. Our AgNW-IZO composite TCFs featured a sheet resistance (R_s_) of approximately 3.3 ohm/sq with a transparency of 72% at λ = 550 nm.

## 2. Materials and Methods

### 2.1. Materials

AgNWs were successfully synthesized through a simple hydrothermal process without the use of surfactants or polymeric additives. In a typical synthesis, 4.5 mL of deionized (DI) water containing 0.02 mol of silver nitrate (Aldrich, St. Louis, MO, USA) and 3 mL of DI water containing 0.02 mol of sodium chloride were added dropwise and simultaneously to 7 mL of DI water under magnetic stirring to form a colloidal silver chloride suspension. Glucose (0.012 g) was then dissolved in the suspension, which was subsequently transferred to a stainless-steel autoclave with a Teflon liner. The reaction mixture was heated at 160 °C for 18 h under autogenous pressure. After reaction, the mixture was washed with absolute ethanol and centrifuged at 3000 rpm for 20 min. The purification procedure was repeated several times until the AgNWs were completely separated from particles.

The colloidal precursor for IZO was prepared by dissolving 0.2 mol of zinc acetate [Zn(CH_3_COO)_2_] and 0.2 mol of indium nitrate [In(NO_3_)_3_] in 5 mL of methyl glycol. The mixture was injected into a flask heated by an oil bath at 150 °C under magnetic stirring. Subsequently, 1.2 mL of ethanolamine and 300 µL of glacial acetic acid were added sequentially to the precursor solution. The reaction mixture was further refluxed at 150 °C for 1 h and then allowed to cool to room temperature, followed by aging for 24 h.

### 2.2. Preparation of Composite Films

The AgNWs were dispersed in isopropyl alcohol (IPA) and deposited onto glass substrates via a drop-casting method. The randomly distributed AgNW meshes were annealed at 400 °C for 30 min to reduce the contact resistance between AgNWs. Subsequently, an IZO colloidal solution was spin-coated onto the pre-deposited AgNW meshes at 2400 rpm for 60 s. The AgNW-IZO composite film was then annealed in a tube furnace at 450 °C for 1 h to remove residual organic components.

### 2.3. Characterization

The crystal structure of the pure AgNW meshes was examined by a X-ray diffraction (XRD) spectrometer with Cu Kα radiation (0.15418 nm) (D/max-RA, Rigaku, Tokyo, Japan). The microstructures of the AgNW networks and the AgNW-IZO composite films were observed with a field emission scanning electron microscope (SEM) (Quanta FEG 250, FEI, Hillsborough, NC, USA). Transmittance spectra were acquired with a UV/Vis/NIR spectrophotometer (Lambda 900, PerkinElmer, Waltham, MA, USA). The sheet resistances of the conductive films were measured by the four-probe technique with a precision current source and a nanovoltmeter (Keithley 6220 and Keithley 2182A, Keithley, Cleveland, OH, USA).

## 3. Results and Discussion

### 3.1. Structural Properties and Surface Morphology

**Figure 1:**
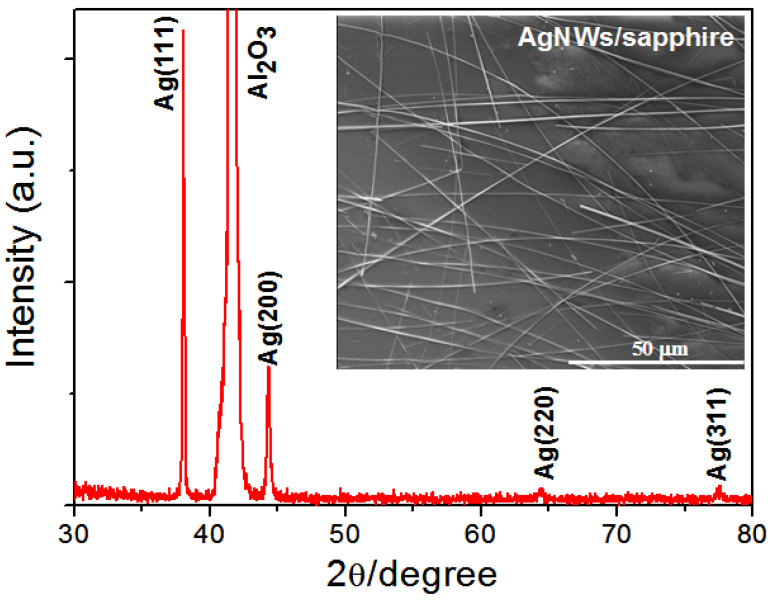
XRD pattern of the as-prepared AgNWs. The inset shows a SEM image of the uniformly distributed AgNWs.

Figure 1 presents XRD patterns of AgNWs prepared by drop-casting onto a sapphire substrate. All the diffraction peaks can be indexed to the face-centered cubic phase of silver. The calculated lattice constant is 4.0952 Å, which is close to the standard value (a = 4.0862 Å, JCPDS Card File No. 04-0783). The inset shows a SEM image of the AgNWs with diameters of 300 nm and lengths ranging from 60 to 150 µm. The density of the AgNW mesh can be simply varied by changing the concentration of the AgNW dispersion. The high aspect ratios of AgNWs are comparable to those of materials employed as high-performance transparent conductors in studies reported by Winey’s group [27].

**Figure 2:**
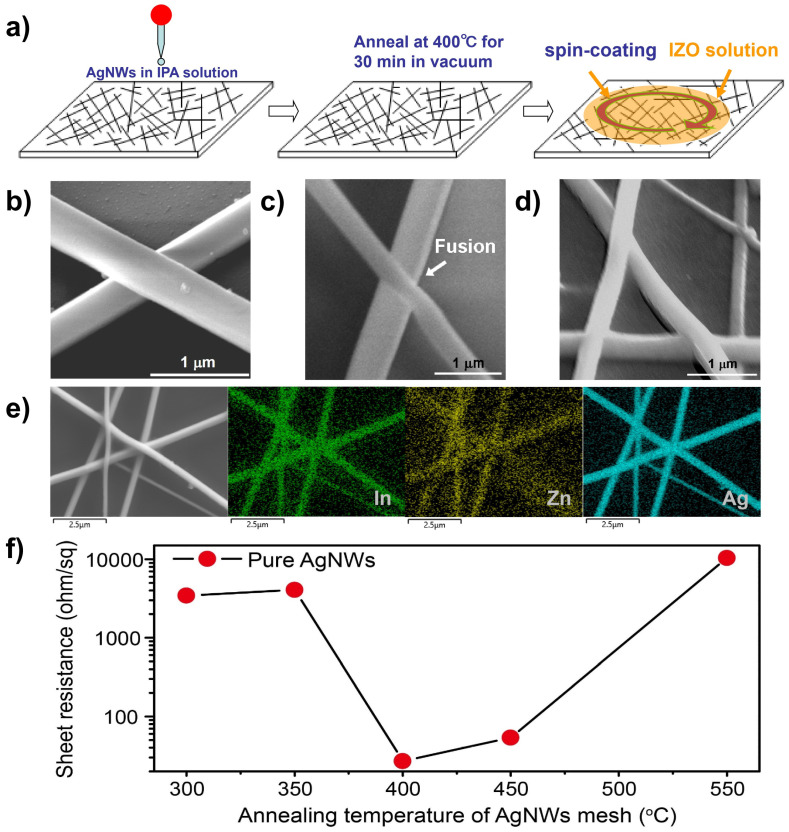
(**a**) Schematic representation of the fabrication process of AgNW-IZO composite films. SEM images of: (**b**) pure AgNW meshes deposited onto glass substrates without futher treatment, (**c**) after annealing, and (**d**) after incorporation of inorganic IZO into the AgNW mesh. The IZO nanostructures are localized near the AgNW junctions. (**e**) SEM image and EDS element mapping of the IZO within the composite film. (**f**) Variation in sheet resistance values for pure AgNW mesh films as a function of temperature under vacuum conditions.

The fabrication process of the AgNW-IZO composite electrodes is illustrated in Figure 2a. Corresponding SEM images acquired at each processing step are presented in Figure 2b–d. Following the first step, a silver mesh film was formed, with AgNWs randomly distributed across the substrate. The junction between two AgNWs is shown in Figure 2b. Due to the AgNWs being connected solely by gravitational settling and weak van der Waals interactions, the contact area between AgNWs is limited, resulting in a R_s_ exceeding 4000 ohm/sq for the AgNW mesh. Furthermore, the adhesion of the AgNWs to the substrate was insufficient to withstand subsequent processing steps. In the second step, annealing was carried out at 400 °C for 30 min under a vacuum of 10^−1^ Pa to reduce the electrical resistance at the nanowire junctions and enhance the adhesion of the AgNW meshes to the substrates. After this process, the R_s_ of the mesh films decreased to 27 ohm/sq. As shown in Figure 2c, the AgNWs melted during heating and fused at the junctions. The SEM image and energy-dispersive X-ray spectroscopy (EDS) elemental mapping of the IZO coating within the composite film are presented in Figure 2e. It was found that the indium and zinc elements are evenly distributed on the Ag NW surface and throughout the surrounding mesh structure. Figure 2f illustrates the dependence of the Rs of AgNW mesh films on the annealing temperature. In the third step, an IZO colloidal solution was deposited onto the annealed AgNW meshes via spin-coating. To remove residual organic components, the AgNW-IZO composite films were annealed at 450 °C for 1 h in air. The resulting films exhibited a R_s_ of approximately 3.3 ohm/sq. Furthermore, the composite films demonstrated enhanced thermal and thermo-oxidative stability, as will be elaborated upon in subsequent sections.

### 3.2. Electrical and Optical Properties

**Figure 3:**
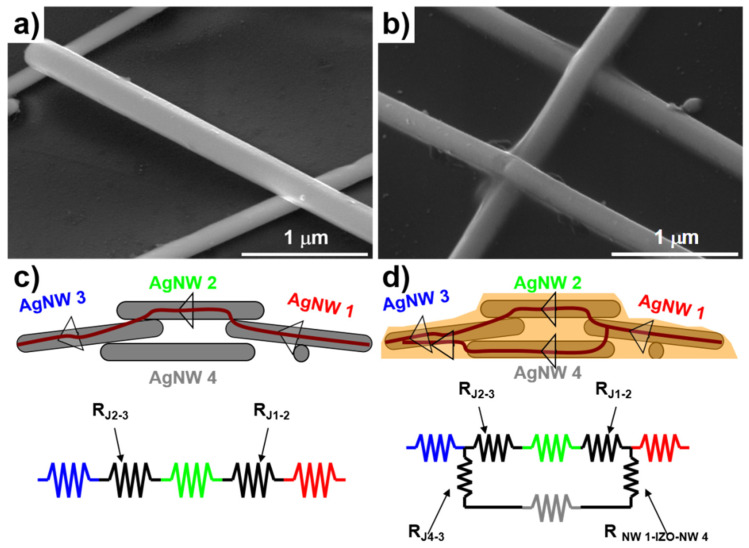
(**a**,**c**) SEM images of the junction between two nanowires; (**b**,**d**) schematic diagrams of resistance circuits for pure AgNW mesh and AgNW-IZO composite films, respectively.

The junction resistance between individual AgNWs constitutes a limiting factor for the R_s_ of AgNW-based TCFs. Figure 3a presents an SEM image of a pristine AgNW mesh, in which the contact area of the adjacent AgNW junction is apparently small. In pure AgNW networks, the R_s_ is primarily determined by the junction resistance (R_J1–2_ and R_J2–3_), given the inherently low resistivity of silver metal. The following equation is employed to quantify the contribution of junction resistance to the total R_s_ of AgNW-based TCFs.

(1) $$R_{s}=R_{NW1}+R_{J1-2}+R_{NW2}+R_{J2-3}+R_{NW3}$$

A resistance circuit schematic diagram (Figure 3c) for the pristine AgNW meshes system was used to qualitatively model the electron transport pathways through the wires. In the composite films (Figure 3b), the conductive IZO particles fill the empty regions in the AgNW mesh, thereby providing additional electron transport pathways and reducing the junction resistance between adjacent AgNWs. As a result, the R_s_ of the composite films was one order of magnitude lower than that of the AgNW network alone and two orders of magnitude lower than that of the IZO films. The electron transport pathways in the composite film are illustrated in the schematic diagram shown in Figure 3d. In the composite films, the R_s_ arises from the resistances of the individual components as well as their contact resistances (R_NW1-IZO-NW4_). The sheet resistance of the composite (R_s-composite_) can be modeled as follows:

(2) $$R_{s\text{-}composite}=R_{NW1}+R^{\prime }+R_{NW3}$$

where R′ is the parallel resistance of the resistance circuit schematic, determined from:

(3) $$R\prime =\frac{\left(R_{J1-2}+R_{NW2}+R_{J2-3}\right)\left(R_{NW1\text{-}IZO\text{-}NW4}+R_{NW4}+R_{J4-3}\right)}{\left(R_{J1-2}+R_{NW2}+R_{J2-3}\right)+\left(R_{NW1\text{-}IZO\text{-}NW4}+R_{NW4}+R_{J4-3}\right)}$$

Therefore, the conductivity of the AgNW-IZO composite films is enhanced through the provision of additional conductive pathways and an increased contact area for the AgNWs.

Surface morphology and mechanical adhesion represent critical considerations for AgNW-based TCFs. The composite films exhibit a smoother surface compared to the AgNW networks alone, as evidenced in Figure 3b, due to the filling of empty regions on the substrate. The voids between the AgNW mesh and the glass substrate were filled with colloidal IZO. The IZO coating functions as a protective layer on the randomly distributed AgNW mesh, thereby enhancing the mechanical cohesion of the hybrid structure. The IZO coating after annealing treatment exhibited an optical transmittance value of 98.2% at 550 nm and a corresponding R_s_ value of approximately 8.0 × 10^4^ ohm/sq.

**Figure 4:**
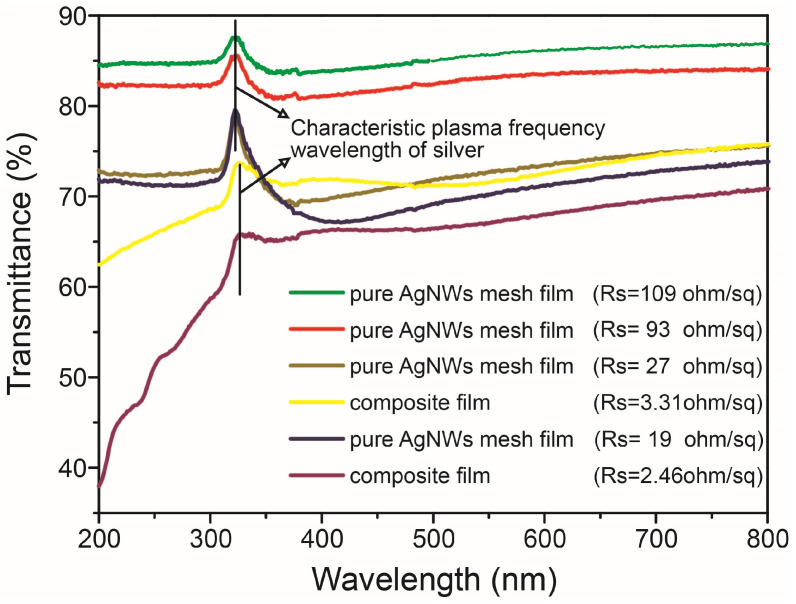
Optical transmittance spectra of pure AgNW mesh films: Sample 1 (green), Sample 2 (red), Sample 3 (dark yellow), Sample 4 (navy), and composite films C1 (yellow), C2 (purple).

Figure 4 presents the transmittance spectra for the pure AgNW mesh film and AgNW-IZO composite films with several different nanowire densities. The optical transparency of the AgNW meshes decreases as the nanowire density increase. The random meshes of the AgNWs exhibited optical transmittance values at 550 nm (T) of 85.6%, 83.1%, 72.7%, and 70.2% with corresponding R_s_ values of 109, 93, 27, and 19 ohm/sq for Samples 1–4, respectively. The pure AgNW meshes showed high optical transparency across all the entire measured wavelength range, which can be attributed to the predominant influence of mesh sparseness on light transmission through the randomly distributed nanowires. Notably, a transmission maximum was observed at 322 nm in the spectra, which corresponds to the characteristic plasma frequency of silver in the pure AgNW meshes [28]. The AgNW-IZO composite film (C1) exhibited approximately 1% lower transmittance compared to the pure AgNW mesh film (Sample 3), attributable to light absorption by the small amount of IZO colloid present in the interstitial regions of the AgNW mesh. The rapid decrease in transmittance of the composite film below the characteristic plasma frequency wavelength of silver is attributed to the band-to-band absorption in the IZO coating. However, the R_s_ of the composite film (C1) was approximately 13% lower than that of Sample 3. Transparent electrodes require both low R_s_ and high optical transparency. Therefore, the optical transmittance (T) at 550 nm and the R_s_ were employed to calculate the figure-of-merit (Φ_TE_) for the transparent conducting electrodes, as defined by Haacke [29].

(4) $$\Phi_{TE}=\frac{T^{10}}{R_{S}}$$

To compare the pure AgNW mesh films with composite structures, we plotted Φ_TE_ for TCFs with two AgNW densities, as listed in Table 1. The optimum value of Φ_TE_, as determined from the plot, was achieved by the composite film (C1) at 10.645 × 10^−3^ ohm^−1^, which was approximately 7 times higher than that of the pure AgNW mesh film (Sample 3, 1.529 × 10^−3^ ohm^−1^). The increase in conductivity can be attributed to the presence of the inorganic conducting material (IZO) located between the nanowires and to enlarged contact area of AgNWs. It is particularly noteworthy that such a high Φ_TE_ value can be achieved through a simple spin-coating process. At the higher AgNW density, the Φ_TE_ of the AgNW-IZO composite film (C2, ~7.49 × 10^−3^ ohm^−1^) was approximately 5 times as high as that of the pure AgNW mesh film (Sample 4, ~1.532 × 10^−3^ ohm^−1^). Furthermore, the Φ_TE_ value of the composite film (C1) exceeded that of a conventional TCF deposited from a traditional material such as ITO. In the transmittance spectra (Figure 4), it is also observed that the characteristic plasma frequency wavelength of silver increases from 321 to 326 nm when the pristine AgNW network (Sample 3) forms a composite film (C1). The optical properties of nanoparticles are influenced by variations in their size, shape, and dielectric environment [30]. The dielectric environment can be characterized by the interaction between the refractive indices of the nanowires and their surrounding medium. Hence, we assumed that the changes in the refractive index of the material surrounding the AgNW mesh influence its optical properties. The shift in plasma frequency wavelength primarily arises from the increase in refractive index when transitioning from IZO to air in the AgNW-IZO composite film.

**Figure 5:**
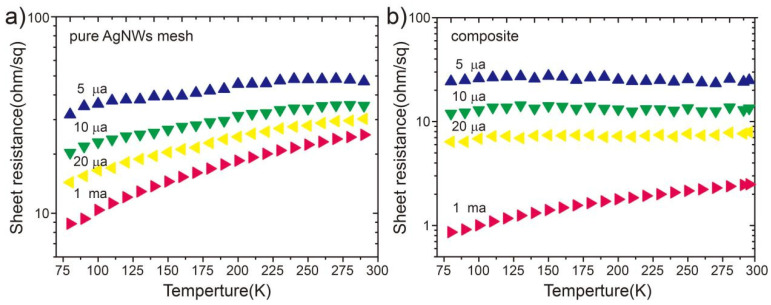
Temperature dependence of R_s_ for pure AgNW mesh film (**a**) and composite film (**b**). Measured output current using a constant source: 5 µA (blue triangles), 10 µA (green triangles), 20 µA (yellow triangles), and 1 mA (pink triangles).

A notably high Φ_TE_ value for the composite film (C1) sample suggests that the charge transport mechanisms of the pure AgNW mesh and composite film are fundamentally different. To clarify this discrepancy, the temperature dependence of R_s_ was measured for both samples. Figure 5a shows the temperature dependence of R_s_ in the range 80–290 K for the pure AgNW mesh film. As shown in Figure 5a, the R_s_ value of the pure AgNW mesh film increases linearly with increasing temperature under different constant current outputs (5, 10, and 20 µA, and 1 mA). This behavior is typical of electronic transport in metals. For metallic resistance, R_0_ denotes the resistance at 0 °C (T_0_) and α represents the temperature coefficient of resistance used to calculate R(T) as a function of the temperature change, according to [31]:

(5) $$R\left(T\right)=R_{0}\left[1+\alpha \left(T-T_{0}\right)\right]$$

Conversely, the R_s_ values of the AgNW-IZO composite film exhibit a nonlinear trend with increasing temperature at lower output currents (i.e., 5, 10, and 20 µA), as shown in Figure 5b. It is concluded that the observed waveform originates from the presence of a potential difference that can partially impede the flow of smaller currents. Therefore, as temperature increases, the change in R_s_ depends on both the metallic material (AgNWs) in the conductive pathway and the semiconductor (IZO). Under an output current of 1 mA, the R_s_ of the composite film exhibits a linear increase with increasing temperature. This result suggests that a slightly higher current is not affected by the potential barrier formed at the interfaces of AgNW-IZO composite structures. Electrons with sufficient energy can overcome the potential barrier and move freely through the layer. This phenomenon further indicates that inorganic IZO actively participates in conduction alongside the AgNW meshes, serving as a conductive component.

### 3.3. Material Stability

**Figure 6:**
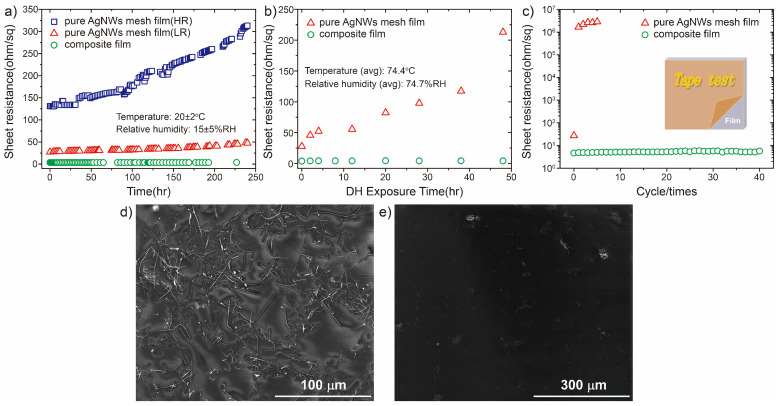
Evolution of sheet resistance over time under ambient conditions (**a**) and DH conditions (**b**): pure AgNW mesh film (HR) (blue squares), pure AgNW mesh film (LR) (red triangles), and AgNW-IZO composite film (green circles). (**c**) Changes in R_s_ during tape adhesion test for both films. SEM images of the adhesive tape after peeling test for the pure AgNW mesh film (**d**) and composite film (**e**).

Stability against oxidation and mechanical adhesion are critical factors for the application of AgNW-based TCFs. The thermal oxidation stability and mechanical adhesion performance of both pure AgNW mesh and composite films were further evaluated. As shown in Figure 6a, the as-deposited test samples were placed in a controlled environment maintained at 20 ± 2 °C and relative humidity 15 ± 5% for 240 h. The R_s_ of both high-resistance (HR) low-resistance (LR) AgNW mesh films increased by a factor of two after 240 h. Conversely, the R_s_ of the AgNW-IZO composite film exhibited only a 3.66% increase under the same conditions. The R_s_ value stabilized at 3.68 ohm/sq (from approximately 3.3 ohm/sq) after 8 months. The long-term oxidation stability of the composite films has benefits for long-term performance in TCF applications. The R_s_ values of the pure AgNW mesh and composite films were compared under damp heat (DH) conditions (temperature: ~74.4 °C and relative humidity: ~74.7%) for 48 h, as shown in Figure 6b. The R_s_ value of the pure AgNW meshes increased rapidly upon exposure to water at elevated temperatures. However, as observed previously, the R_s_ value of the composite film increased slightly to 5.77% owing to the protective layer around the AgNW mesh. The reliability of this composite film represents a valuable attribute for its application in optoelectronic devices.

Currently, poor adhesion to the substrate remains a critical limitation that hinders the application of pure AgNW meshes in electronic devices. The proposed fabrication method for AgNW-IZO composite films effectively addresses this issue. IZO nanoparticle structures filled the gaps between the AgNWs and the substrate, thereby enhancing the overall adhesion of the structure to the substrate. Figure 6c shows the change in R_s_ for both the pure AgNW mesh and the composite films over 40 tape test cycles. The pure AgNW meshes were readily removed from the substrate using adhesive tape (see Figure 6d), due to their weak interfacial adhesion. Consequently, the R_s_ of the film increased dramatically after the second cycle. In contrast, the AgNW-IZO composite structure exhibited excellent stability. Few AgNWs were observed on the adhesive tape (see Figure 6e), and those present were considerably smaller than the average size. Notable, the composite film exhibited high tolerance to the tape test and maintained stable R_s_ over 40 cycles. This robust stability may help address critical challenges in the fabrication of optoelectronic devices based on metal nanowires.

**Figure 7:**
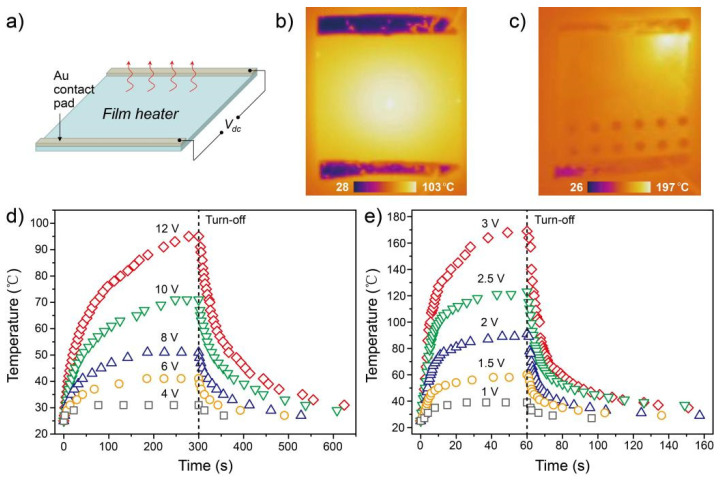
(**a**) Schematic illustration of the configuration of a film heater. Infrared images of the ITO (**b**) and AgNW-IZO (**c**) film heaters at 12 and 3 V, respectively. Temperature-time profiles of the ITO (**d**) and AgNW-IZO (**e**) film heaters under different applied voltages.

To demonstrate the potential applicability of the AgNW-IZO composite film (C1) as a transparent heater, a DC voltage was applied to the heater through gold contact pads located at the film edges, as schematically illustrated in Figure 7a. Figure 7b,c show infrared images of the ITO and AgNW-IZO composite films, respectively, recorded using an IR camera at applied voltages of 12 and 3 V. Figure 7c shows a uniform heat distribution over the AgNW-IZO composite film, which suggests that a uniform dispersal of AgNWs in the mesh was achieved during film fabrication. In Figure 7d,e, the temperature–time profiles of the ITO and the AgNW-IZO composite films were recorded as a function of applied DC bias voltage. As shown in Figure 7d, the ITO heater exhibited a negligible temperature increase at an applied voltage of 4 V, whereas the temperature rose to 95 °C when the applied voltage was increased to 12 V. In contrast, the AgNW-IZO composite film heater achieved a temperature of approximately 40 °C at an applied voltage of 1 V. When the applied voltage was increased to 3 V, the heater reached approximately 170 °C, demonstrating effective operation at low applied voltages. The temperature changes were observed to occur more rapidly in the AgNW-IZO composite film than in the ITO. This result can be attributed to the higher thermal conductivity of metallic silver compared to that of semiconductor materials. Therefore, AgNW-IZO composite films exhibit promising potential for use as transparent heating elements.

## 4. Conclusions

In conclusion, we propose a composite structure prepared from a mesh of AgNWs and an IZO sol–gel, for the realization of high-performance transparent conductors. By adding IZO nanoparticles to the AgNW mesh, the R_s_ was markedly decreased (to approximately 13% of the original value) withoutsacrificing transparency. A R_s_ of 3.3 ohm/sq was obtained at a transparency of 72% (at λ = 550 nm) in this composite film. Furthermore, the composite structure showed excellent stability under an oxidation test with a stable Rs of 3.68 ohm/sq after 8 months and only a 5.77% increase in Rs after DH aging and mechanical adhesion tape tests. The electrical properties of the composite films were also investigated under various output currents. The inorganic IZO functions as both a protective and adhesive layer, as well as providing additional conductive pathways in the AgNW mesh. This hybrid structure might improve the performance of AgNW-based TCFs. Furthermore, the AgNW-IZO composite films demonstrate promising applicability as transparent heating elements.

## Figures and Tables

**Table 1:** Comparison of the R_s_, optical transmittance, and figure-of-merit Φ_TE_ for pure AgNW mesh films (Samples 3 and 4), composite films (C1, C2), and a sputtered ITO film.

Sample	Sheet Resistance (ohm/sq)	Transmittance (%) at 550 nm	Figure of Merit (10^−3^ ohm^−1^)
AgNWs mesh film Sample 3	27	72.7	1.529
AgNWs-IZO composite film C1	3.3	71.5	10.645
Pure AgNWs mesh film Sample 4	19	70.2	1.532
AgNWs-IZO composite film C1	2.46	67.1	7.490
Sputtered ITO	40	90.5	9.210

## Data Availability

The original contributions presented in this study are included in the article. Further inquiries can be directed to the corresponding author.
